# Correlating nuclear morphometric patterns with estrogen receptor status in breast cancer pathologic specimens

**DOI:** 10.1038/s41523-018-0084-4

**Published:** 2018-09-04

**Authors:** Rishi R. Rawat, Daniel Ruderman, Paul Macklin, David L. Rimm, David B. Agus

**Affiliations:** 1 Lawrence J. Ellison Institute for Transformative Medicine, University of Southern California, 2250 Alcazar Street, CSC 240, Los Angeles, CA 90089-9075 USA; 2 Intelligent Systems Engineering, Indiana University, 700N. Woodlawn Ave., Bloomington, IN 47408 USA; 3 Department of Pathology, BML 116, Yale University School of Medicine, 310 Cedar St, PO Box 208023, New Haven, CT 06520-8023 USA

## Abstract

In this pilot study, we introduce a machine learning framework to identify relationships between cancer tissue morphology and hormone receptor pathway activation in breast cancer pathology hematoxylin and eosin (H&E)-stained samples. As a proof-of-concept, we focus on predicting clinical estrogen receptor (ER) status—defined as greater than one percent of cells positive for estrogen receptor by immunohistochemistry staining—from spatial arrangement of nuclear features. Our learning pipeline segments nuclei from H&E images, extracts their position, shape and orientation descriptors, and then passes them to a deep neural network to predict ER status. After training on 57 tissue cores of invasive ductal carcinoma (IDC), our pipeline predicted ER status in an independent test set of patient samples (AUC ROC = 0.72, 95%CI = 0.55–0.89, *n* = 56). This proof of concept shows that machine-derived descriptors of morphologic histology patterns can be correlated to signaling pathway status. Unlike other deep learning approaches to pathology, our system uses deep neural networks to learn spatial relationships between pre-defined biological features, which improves the interpretability of the system and sheds light on the features the neural network uses to predict ER status. Future studies will correlate morphometry to quantitative measures of estrogen receptor status and, ultimately response to hormonal therapy.

## Digital pathology: Hormone receptor status predicted from pathology slides

An artificial intelligence tool that analyzes the morphology of cell nuclei can help pathologists predict whether a breast cancer sample expresses the estrogen receptor (ER) or not. David Agus from the University of Southern California in Los Angeles, USA, and colleagues designed a machine-learning algorithm to correlate ER status — which is usually determined via immunohistochemistry assays — with visual patterns of shape, orientation and other nuclear features that a pathologist normally sees on a stained biopsy specimen. The researchers trained the algorithm on samples taken from 57 women with untreated invasive ductal carcinoma. They then tested the model’s accuracy on a separate set of 56 patient samples. The algorithm could predict ER status with reasonable precision and accuracy, suggesting that, with improvements, it could form the basis of a diagnostic aid for guiding treatment decisions.

## Introduction

Machine vision holds the promise to transform solid tumor pathology. It can correct variations in stain intensity that bias interpretation, calculate correlations between tissue morphology and outcome, and quantify stromal features that are not traditionally studied. For example, Beck et al. used machine vision to identify stromal correlates to breast cancer outcomes,^1^ and the recent CAMELYON challenges showcase the power of machine vision for detection tasks within pathology.^2^ A natural next step for machine-defined morphometrics is to demonstrate the potential to define visual features that correlate to molecular markers, or biologic pathway activation.

It is critical to characterize growth receptor pathways in breast cancer via hormone receptor and HER2 status for patient management in breast cancer. In the US, the standard of care uses multiple immunohistochemistry (IHC) stains for estrogen receptor (ER), progesterone receptor (PR), and HER2 to categorize the breast tumor, determine prognosis and select treatment regimens.^3,4^ However, these assays may be inconsistent across laboratories,^5^ and they are somewhat expensive and often challenging in low resourced settings. However, the marker status is one of the oldest companion diagnostic tests, even though it has relatively low sensitivity and specificity.^3,6^ For example, only 50% of women with ER-positive tumors and 60–70% of women with ER-positive and PR-positive tumors show partial or complete response to tamoxifen therapy.^7–9^ While pathologists have long seen a correlation between low grade morphology and ER+ status, new developments raise the possibility that quantitative deep-learning based morphology may be able to predict molecular ER status, or perhaps even response to hormonal therapy. In this pilot study, we explored how deep learning on H&E-based morphometric features could distinguish ER-negative breast cancer from ER-positive cancer.

## Results

### Nuclear morphometric features predict ER status

We obtained publicly available H&E images and corresponding clinical ER status (positive/negative, determined by IHC) for a tissue microarray of 131 treatment-naïve invasive ductal carcinoma (IDC) patients^10^ (Table 1). After segmenting nuclei and applying a quality control step to exclude over-segmented images (Supplemental Figure 1), we randomized images into a training set (57 patients) and a test set (56 patients). We extracted nuclear morphometric features (shape and orientation) from each nucleus in the training set and fed these measurements into a deep convolutional neural network to learn spatial patterns that correlate to ER-positive or ER-negative status. The DNN was designed to produce a spatial heatmap of ER-positive or negative status. When an input image is fed into the DNN, the output is a heatmap of predictions where intense regions correspond to ER-negative status. The overall ER-status prediction for a patient is the average value of the heatmap.

**Table 1:** Summary of data

Descriptor	Value
Dataset name	IDC
Source	Biomax.us, BM140-sur01^11^
Total patients*	140
Number of patients with known ER status	131
Patients excluded via quality control step	18
Patients after Quality Control Step	113
Grade I	8
Grade I–II	21
Grade II	79
Grade II–III	4
Grade III	1
Number of patients in train set	57
Number of images in train set	57
Number of patients in test set	56
Number of images in test set	56
Approx. image size (pixels)	3000 × 3000
Resolution	20 × (0.5 µm/pixel)

**Fig. 1:**
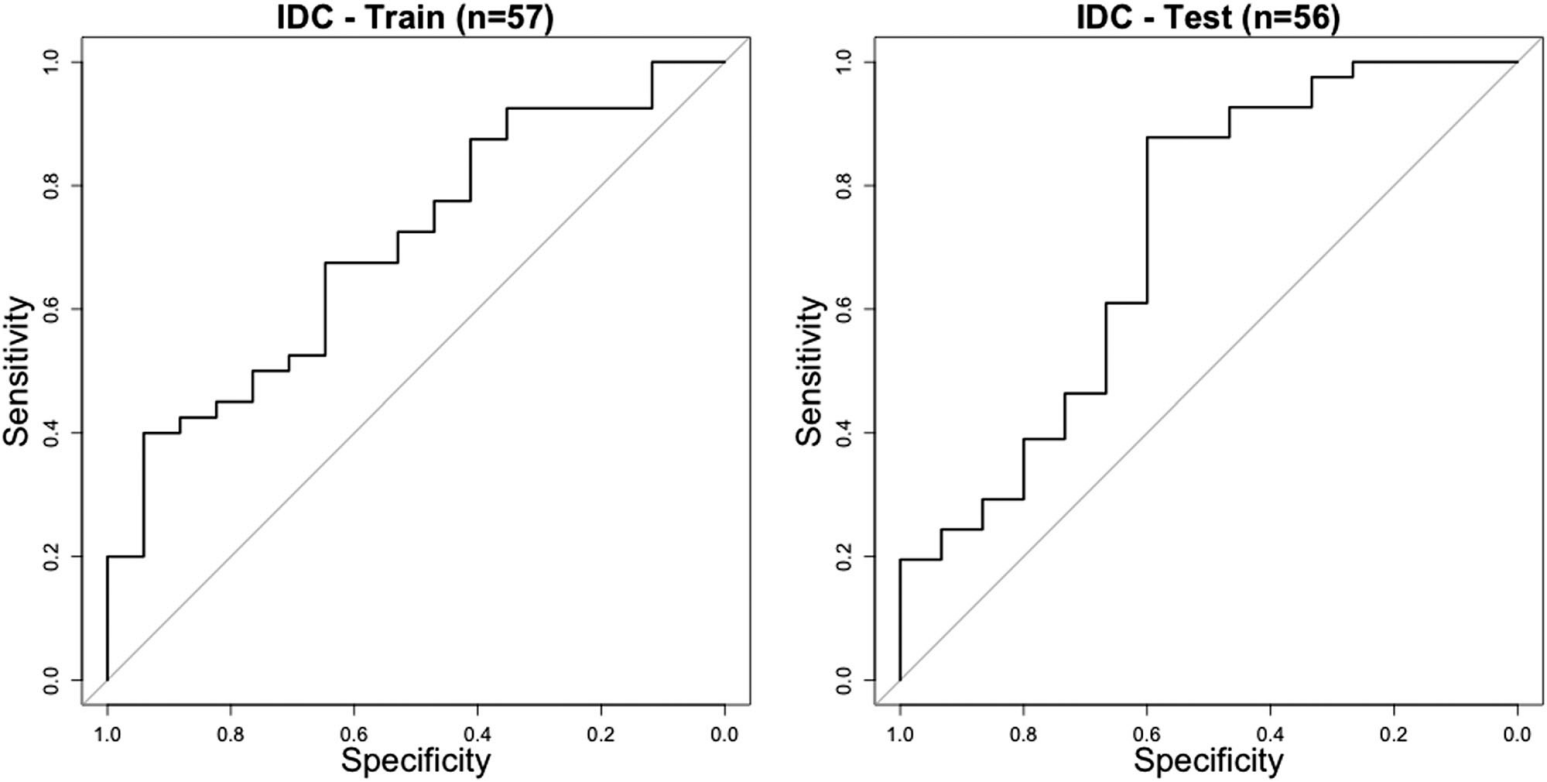
Receiver operating characteristic (ROC) curves for the training dataset (AUC = 0.70, 95%CI = 0.56–0.85) (left), and test dataset (AUC = 0.72, 95%CI = 0.55–0.89) (right)

After training the neural network, we tested the pipeline on the test set and measured area under the receiver operating characteristic curve (AUC) scores of 0.70 (95%CI = 0.56–0.85) and 0.72 (95%CI = 0.55–0.89) on the training and test sets, respectively (Fig. 1). This result suggests our pipeline learned to predict ER status significantly. The similarity between the AUC scores on the training and test sets suggests that the pipeline is not overfitting the training data (in such a scenario training AUC would be significantly higher than test AUC), and that it generalizes well to unseen data.

### A correlation between nuclear size, heterogeneity, and ER status

While deep networks are typically considered to be uninterpretable “black boxes,” we applied several techniques to reverse-engineer the system and understand the morphometric patterns the DNN used to classify ER status. Our first step was to visualize the heatmap the DNN learned to predict. This analysis is similar to laying an IHC image over an H&E image; however, while an IHC image shows the real protein expression, the DNN heatmap shows regions estimated by the DNN to be ER-positive or negative. Because the DNN was trained to predict an accurate patient-level classification (not the spatial pattern of ER-staining), the regions predicted on the heatmap may be different from regions predicted by IHC. However, regions on the DNN heatmap contain information that leads to an accurate ER+/− prediction, and are thus diagnostic regions for ER-assessment.

**Fig. 2:**
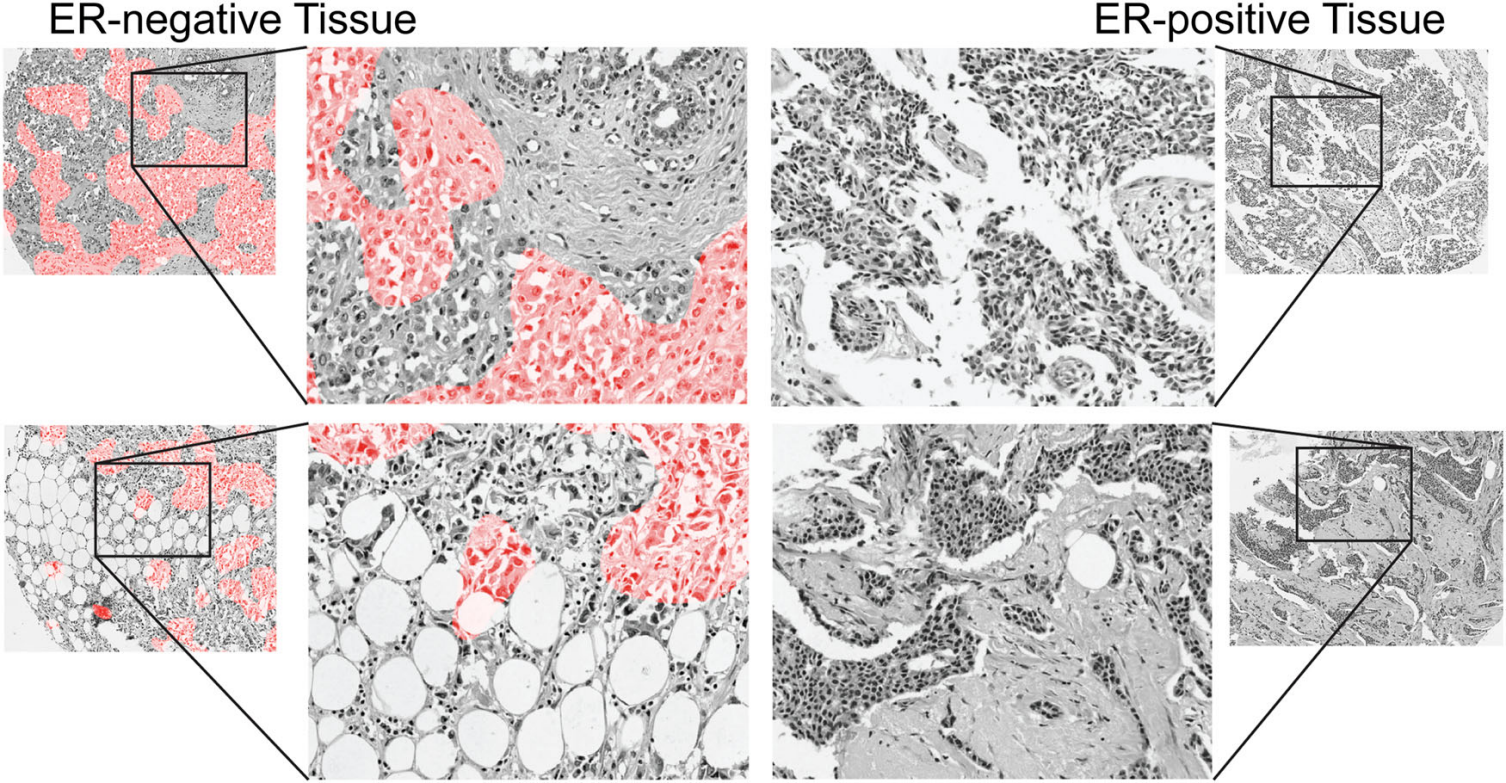
Digital stain for regions predicted to be ER-negative. Pixels are shaded red in regions predicted to be ER-negative with probability greater than 50%. Enlarged regions of ER-negative tissue (left) reveal that the network classifies sub-regions of epithelial tissue as ER-negative. For comparison, ER-positive tissue is shown (right)

For this analysis, we selected several cases that were classified correctly and overlaid the predicted heatmaps on the H&E image to form a “digital stain” where ER-negative regions are colored red and ER-positive regions are uncolored (Fig. 2). By visual inspection, we observed a subset of epithelial areas were predicted ER-negative. Thus, it appears that features in epithelial regions are used by the DNN to classify ER status.

**Fig. 3:**
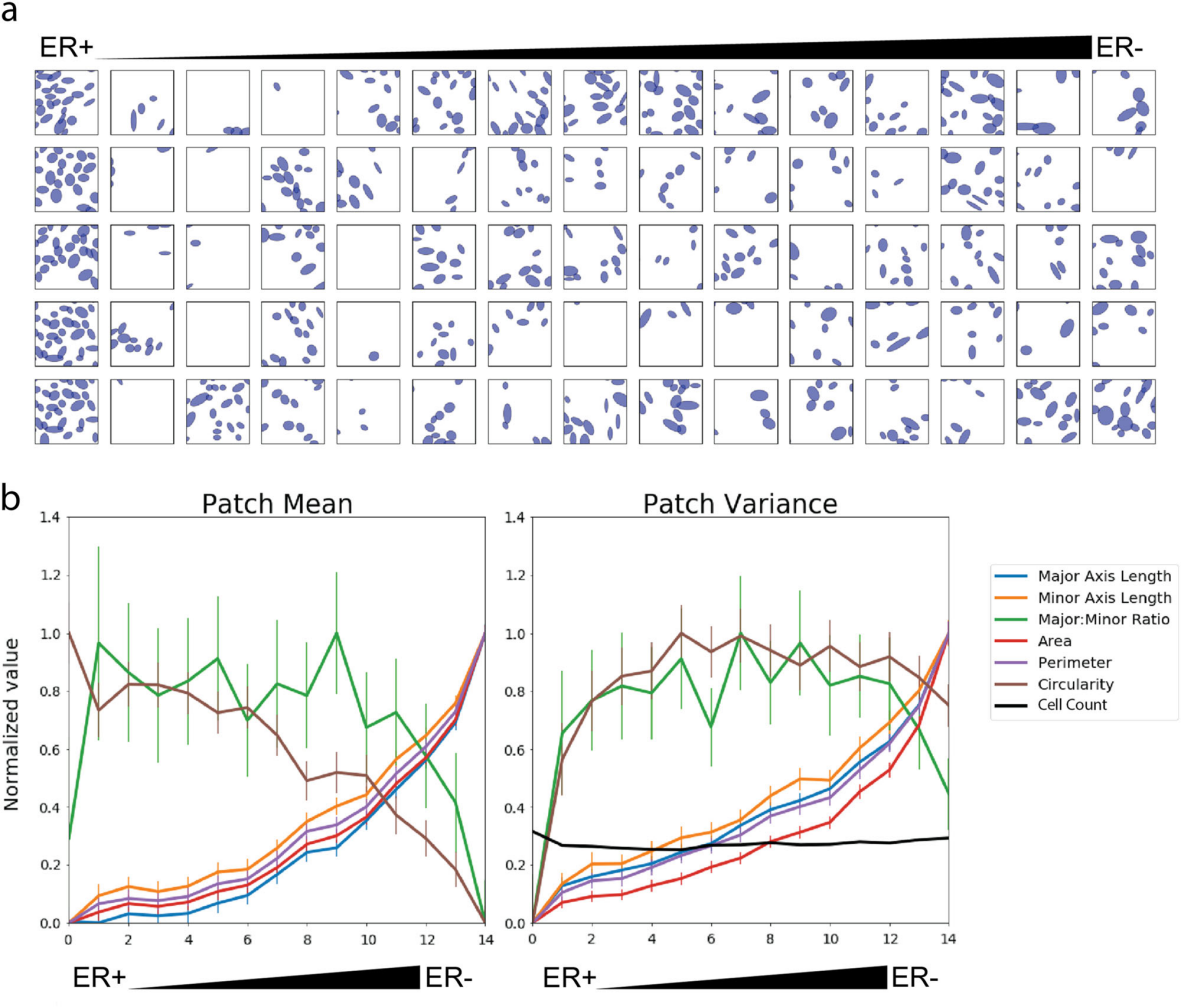
Correlating nuclear morphometric features with ER predictions from the neural network. Image “patches” were extracted from the training dataset, ranked by predicted probability of ER-status, and divided into 15 groups by prediction status. **a** Two representative patches classified as ER positive and ER negative are shown. **b** (Left) The mean of each nuclear feature was calculated within each patch (intra-patch mean); within each group, intra-patch means were averaged to calculate the inter-patch mean. **b** (Right) The variance of each nuclear feature was calculated in each patch (intra-patch variance); within each group, intra-patch variances were averaged. The x-axis in **b** indicates group number, higher group numbers correspond to ER negative predictions

Next, we used the DNN to define spatial parameters related to the specific nuclear features linked to the ER prediction. We divided all of the training images (*n* = 57) into small image patches (64 × 64 pixels, 128 × 128 µm, 11,161 total). Then we predicted the ER score for each patch and sorted the patches by the score from ER-positive to ER-negative. When we looked at the patches most strongly predicted to be ER-positive or ER-negative, we noticed a difference in nuclear size and the variation in nuclear features: ER-negative seemed correlated to larger, more variable nuclei than ER-positive. To formally investigate whether our pipeline learned features related to nuclear size and heterogeneity, we divided the sorted list of image patches into 15 groups ranked by predicted ER score (744 patches per group. Randomly chosen patches from these 15 groups are illustrated in Fig. 3a). For each patch, we calculated the mean value of each nuclear feature (intra-patch mean) and the variance of the feature (intra-patch variance). We also calculated the inter-patch mean and standard error across all patches in each group (Fig. 3b). This revealed that several nuclear morphometric quantities, such as mean height, width, area and perimeter were elevated in patches classified as ER negative. Additionally, nuclear heterogeneity (variance of nuclear features) is correlated to an ER-negative prediction.

Based on these observations, we directly tested if the mean and variance of nuclear features in a patch could predict ER status. We randomly sampled 5000 patches from the training set, calculated the intra-patch means and variances of nuclei within each patch and trained a logistic regression model on these features. Next, we applied the trained logistic regression model to full-sized images in the test set. We divided each image into equally-spaced non-overlapping patches, calculated an ER score for each patch, and averaged the ER score from all patches in each test image. On the training set, we obtained an AUC of 0.648 (95% CI: 0.498–0.799). On the test set, we obtained an AUC of 0.672 (95%CI: 0.494–0.850). While these linear classifiers are less accurate than the DNN, the trend suggests that these features capture information about ER status. Analyzing a DNN trained on expert-defined features helped us interpret the DNN in terms of biological relationships.

## Discussion

We aimed to test feasibility of predicting ER status in breast cancer specimens based on nuclear morphometric features in H&E stained specimens as a way of identifying molecular markers and/or pathway activation without DNA sequencing or other molecular studies. For this pilot study, we define ER-positive by clinical ER status (greater than one percent of cancer cells staining positive for ER on an IHC stain). Using deep learning and labeled tissue images, we trained a learning pipeline to correlate patterns of nuclei to ER status and found that it learned to predict ER with statistical significance. Analysis of the trained model revealed that the network learned an association between large pleomorphic nuclei and ER-negative tumors. While this finding is not novel,^11^ it is significant that this is the first time a neural network learned this relationship without human supervision. As the size of the training dataset grows, we anticipate that it may learn novel patterns not currently recognized in the field. In fact, the ultimate goal of this work would be to evolve to a highly sensitive and specific theragnostic of clinical benefit to hormonal therapy.

A core factor in this work was the development of a hybrid machine-learning approach that combined expert-defined local features with the powerful feature-learning framework of convolutional neural networks. While convolutional neural networks can learn high-order features from the raw image data, training these models typically requires thousands to millions of training images to minimize the impact of noise and color variations. To reduce the impact of stain variation, our study introduced a pre-processing step to extract nuclear morphometric data and developed a novel method for deep learning on these features instead of the raw RGB image pixels. Preprocessing effectively compresses each training image into a vector of morphometric data. While this constrains the types of features the neural network can learn, it also prevents the learning of spurious correlations between nonsensical variables (e.g., staining variation). Thus, we believe using expert-defined features as input allowed the network to learn patterns that generalized well between the training and test datasets.

There are a number of limitations to this work that can be expected in a proof-of-concept study. Most significant is the relatively low AUC achieved, compared to the molecular methods to predict expression of estrogen receptor. We recognize that in this early stage, this test is not close to being a replacement for immunohistochemistry. However, similarly, the best molecular tests for ER status also have a relatively low AUC with respect to prediction of response to hormonal therapy.^12,13^ Furthermore, AUC may not be the best way to evaluate predictive tests, since in treating patients, specificity is always sacrificed for increased sensitivity to prevent any patient from missing the opportunity to benefit from the drug. It is possible that with further effort, deep learning on larger, more comprehensively annotated cohorts will be able to improve the specificity without sacrificing sensitivity.

Another weakness of the work is the relatively small sample size and pilot nature of the study, which focuses on tissue microarray cores. This work focused on the generation of the algorithms and the approach, prior to going through the challenging process of obtaining images from large, comprehensively annotated whole slide images from cooperative group studies. The publication of these pilot studies represents a prerequisite in order to obtain and scan whole sections from the valuable multi-institutional, evidence level 1 trials.

This proof-of-concept demonstrates a technique to correlate morphometric features to a clinical ER receptor status and provides a means to begin understanding the relationships between morphometry and variables of potentially greater clinical significance, such as ER staining heterogeneity or anti-estrogen response. Our hybrid system is not a “black-box” learning system. It learns high-order features based on lower-order, human-defined features that can be reverse-engineered to capture morphologic features that are highly correlated to molecular biology. In this study, we used digital staining and patch analysis to visualize the correlation between large pleomorphic nuclei with ER negative tumors. In future work that incorporates subcellular or extra-cellular features, we can explore how the spatial distribution of nuclei and other features (e.g., nucleoli, mitotic figures, collagen, lymphocytes) correlate to subtypes and outcomes. In fact, the results of the C-Path study^1^ suggest that the information we may extract for the extra-cellular features may be more informative for prediction of response than that cellular features. We believe such algorithms will help researchers understand how the spatial relationships between different types of cells correlate to disease severity and clinical outcomes.

## Method

We hypothesized that the combination of (1) spatial arrangement of cells combined with (2) nuclear morphometric properties would capture important information about the underlying molecular biology of breast cancer and provide clinically useful predictions. Thus, we constructed a learning pipeline to classify cancers by molecular markers. Here, we test this hypothesis on the pathological classification of a tumor as ER+ or ER−. Our method comprises five steps: (1) data acquisition, (2) image pre-processing, (3) quality control, (4) designing and training the neural network, and (5) testing the neural network.

### Step 1: Data acquisition

#### Data

The first set of H&E images we acquired were from the website of the tissue microarray supplier, US Biomax, Inc. (Derwood, MD 20855). As a service to customers, US Biomax, Inc. provides JPEG-compressed H&E images of many tissue microarrays along with immunohistochemistry (IHC) staining information, such as ER receptor status. With permission from US Biomax, Inc., we used the array titled “HBre-Duc140Sur-01” (http://www.biomax.us/tissue-arrays/Breast/HBre-Duc140Sur-01), which contains 140 tissue cores (1.5 mm diameter) from 140 patients diagnosed with invasive ductal carcinoma. We chose this particular microarray because the H&E images displayed minimal staining artifacts and included molecular marker staining status. To collect the data, we used the digital slide viewer on the US Biomax, Inc. website, zoomed in to 20× resolution (0.5 µm per pixel) and took screenshots of each core. These images were correlated to ER status (from the US Biomax, Inc. website), and then fed into the pre-processing pipeline. Following a quality control step (described below), we were left with 113 tissue cores, with one core per patient. We randomly divided these patients into the “Training” (*n* = 57) and “Test” (*n* = 56) datasets.

### Step 2: Image pre-processing

We implemented an automated nuclear segmentation pipeline using Python (version 2.7.12) and Fiji^14^ (version 1.0, a distribution of ImageJ^15^). The steps consist of the following:

1. Scale images as necessary to a resolution 0.5 µm per pixel, using bicubic interpolation.
2. Transform the RGB image into hue, saturation, brightness channels, retaining only the brightness channel for downstream analysis.
3. Apply an automatic, global Otsu threshold^16^ to roughly identify cellular regions.
4. Apply a local adaptive threshold with a radius of 20 pixels (10 µm) to provide fine-scale local separation of nuclei.
5. Use the built-in Fiji watershed transform to separate overlapping nuclei.
6. Calculate the following morphometric parameters for each detected nucleus using the particle analysis functions in ImageJ: center of nucleus (x,y coordinates), major axis length, minor axis length, major axis to minor axis ratio, area, perimeter, and circularity.
7. Convert data into a MultiCellDS digital tissue snapshot (a standardized XML representation for spatial multicellular data)^17^ for storage.

The pre-processing image scripts are available in the supplementary materials. We identified on average 4960 nuclei per image (95% CI = 4650–5270, *n* = 140).

### Step 3: Quality control

We performed a label-blind quality control step in which 200 × 200 pixel patches were extracted from each H&E image and overlaid with ellipses representing the extracted nuclei. Visually, RR assigned a Boolean value (0 or 1) to each image corresponding to whether the image appeared well segmented (defined as greater than 70% concordant, Supplemental Figure 1). Patients with unknown ER status were excluded from the analysis. As a result of the quality control step, we used 113 out of 140 cases.

### Step 4: Designing and training the neural network

**Fig. 4:**
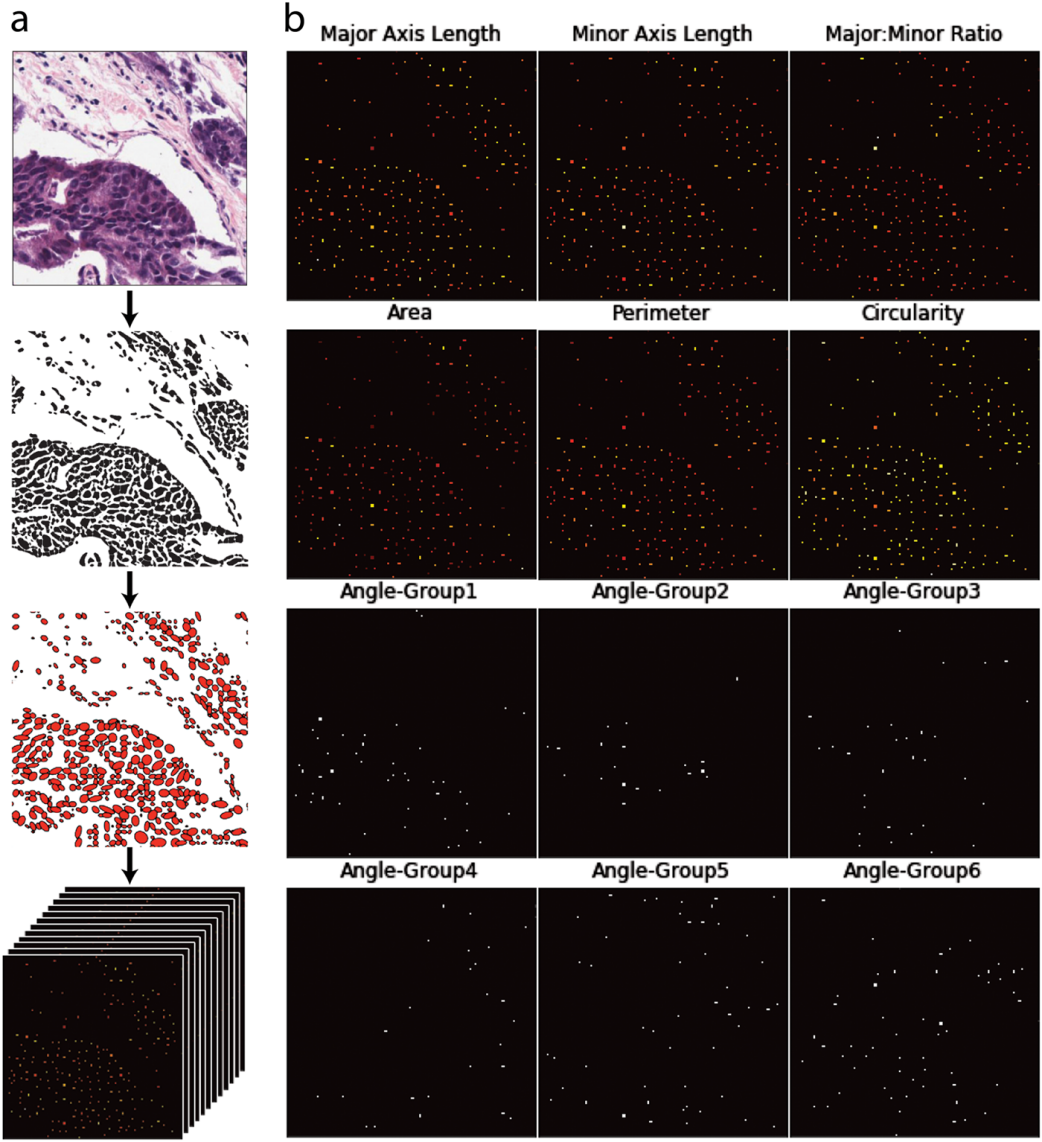
Construction of a sparse 12-channel image. **a** Hematoxylin and eosin-stained tissue are processed by a nuclear segmentation algorithm. Each nuclear feature is measured and represented on a single 2D array, where individual cells are represented as points. Arrays are stacked to form a 12D image. **b** Detailed view of 12 individual channels that would be stacked to form a 12-channel image

We converted each MultiCellDS digital tissue snapshot into a sparse 12 channel image (Fig. 4), consisting of zeros everywhere except at the cell centers, which contain information about the nuclei. The first six channels correspond to cellular shape features (major axis, minor axis, major: minor ratio, area, perimeter, circularity). In addition, we constructed 6 “binary angle” features from the nuclear angle measurement, leading to a total of 12 feature channels; if the major axis of cell *i* has an angle θ_i_ (0 < θ_i_ < 180) with the positive *x*-axis, we define six orientation features φ_i,j_ (1 ≤ j ≤ 6) by

$$\begin{array}{*{20}{l}} {\varphi _{{\mathrm{i}},{\mathrm{j}}} = 1\,{\mathrm{if}}\,30 \times \left( {{\mathrm{j}} - 1} \right) < \theta \_{\mathrm{i}} \le 30 \times {\mathrm{j}}} \hfill \cr {\varphi _{{\mathrm{i}},{\mathrm{j}}} = 0\,{\mathrm{otherwise}}.} \hfill \end{array}$$

The rationale for constructing binary features relates to the training process for the neural network. We wanted the network to learn rotationally invariant features, which are robust to flips and rotations (in the spatial image coordinates) of the 12-D image. Using binary angle channels allowed us to flip or rotate the image while keeping the cell angle information properly oriented.

The final step before training involved downscaling the sparse images 4× via nearest-neighbor scaling to reduce downstream computation. Thus, the DNN sees cell features at a resolution of 2 µm per pixel. Following downsampling, cells positioned at physical coordinates (*x*_1_,*y*_1_), are positioned at matrix indices (*x*_2_,*y*_2_) such that

$$\begin{array}{*{20}{l}} {x_2 = {\mathrm{floor}}\left( {x_1/4} \right)} \hfill \cr {y_2 = {\mathrm{floor}}\left( {y_1/4} \right)} \hfill \end{array}$$

### Network design

**Fig. 5:**
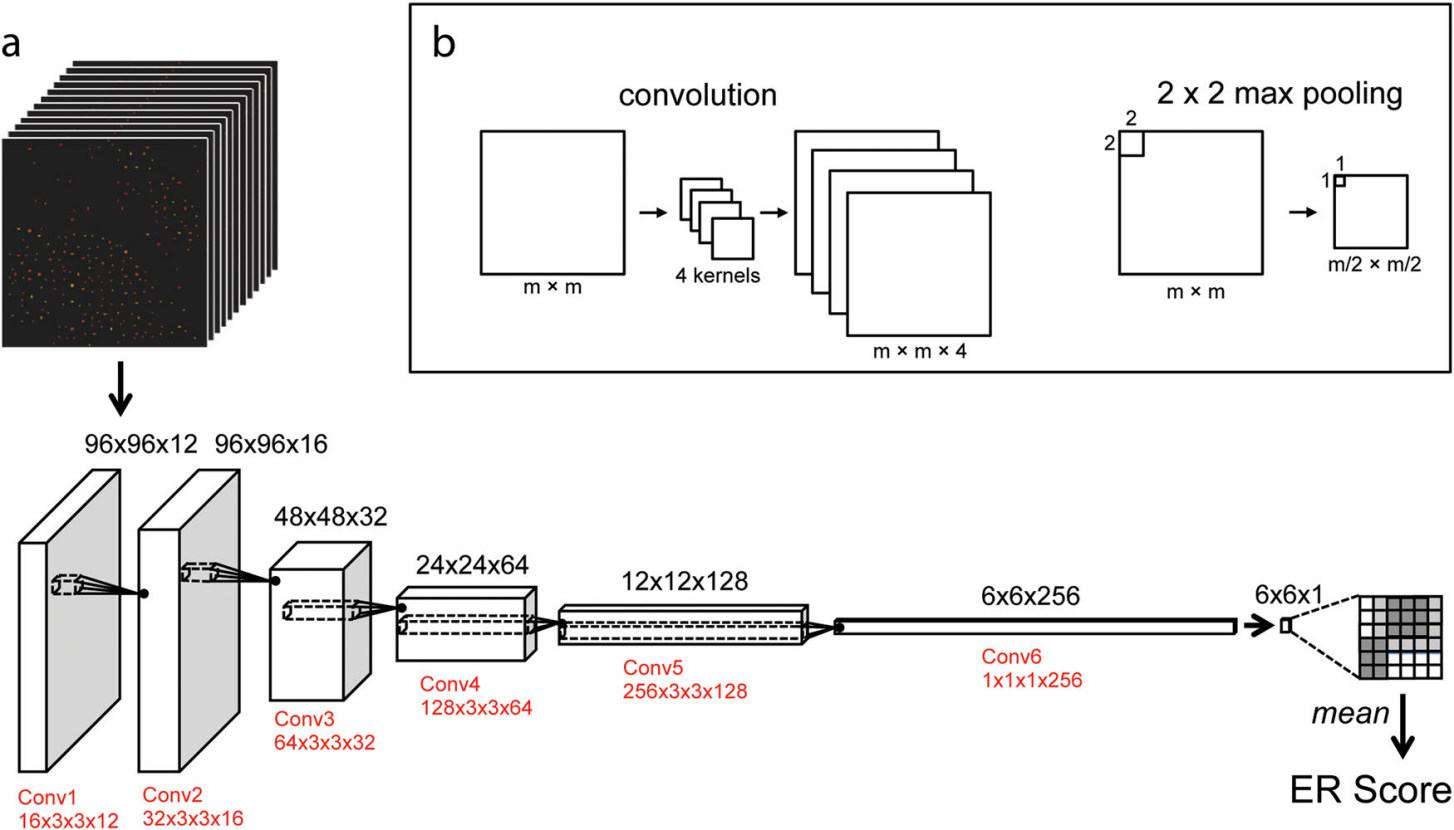
Schematic of the deep neural network. **a** The 12 Channel Image is loaded into a fully convolutional network with six convolutional and max-pooling layers (not shown for simplicity). The output is a 1D map of ER predictions, which is averaged and normalized (not shown) to produce an ER score for the image. The size of the matrix that holds the convolutional weights is indicated in red, where a matrix N × C × X × Y has N Kernels that act on a C channel input of size X × Y × C. **b** An example of convolutional and max pooling operations. In convolution, the starting image (left) is convolved by four kernels (middle) to produce four feature maps (right). In max pooling, the maximum value of each 2 × 2 square is used to produce an output image

The overall structure of our neural network was inspired by previous work applying deep learning to image segmentation^18^ and high-content screening.^19^ Our network has approximately 4.6 × 10^5^ parameters arranged in six fully convolutional layers, 5 max pooling layers, one global mean layer, and one batch-normalization layer (Fig. 5). Through cross-validation on the training set, we decided to use leaky rectifying linear neurons with cross-entropy loss. Importantly, we found that using a batch normalization layer^20^ was necessary for convergence. Over one batch of training data, a batch normalization layer produces outputs with zero mean and unit variance. In training, this leads to a well-distributed set of output predictions, which accelerates the learning process. In addition, we used a dropout layer, which randomly eliminates 50% of the neurons during each round of training to prevent co-adaptation of neurons (a form of over-fitting).^21^

Using a global mean layer gives us the option of training the network on images of arbitrary size. However, we chose to train on small patches extracted from sparse images to increase the relative size of the training set. Thus, during the training process, we randomly extracted small patches (100 × 100 pixels, 200 × 200 µm) from the downscaled feature maps (approx. 750 × 750 pixels, 1500 × 1500 µm) and assigned them the same class as the overall image. At runtime, these patches were randomly flipped and rotated (in multiples of 90 degrees) to augment the dataset and promote the learning of rotationally invariant features. Theoretically, the augmented training set consists of 10^8^ different patches; however only a subset of these images was actually used to train the network.

Each layer in the neural network combines features from the previous layer, and deeper layers can learn higher order features. The model uses a fully convolutional architecture, which means that it can process images of arbitrary size, producing output in the form of a spatial map that scales with the size of the input image.^18^ Thus, the final classification layer produces a spatial map for ER score over the image, and the average prediction over the map is treated as the score for the image.

All experiments were conducted on an Nvidia K80 GPU using the Deep Learning libraries Theano^22^ and Lasagne.^23^

### Network training

We randomly split 113 patients from into training (*n* = 57) and test (*n* = 56) datasets. From the training set, we held out 20% data for cross validation during the training process. From the training set, we subsampled small patches (100 × 100 pixels, 200 × 200 µm) and trained the network using image-level labels (ER+, ER−) for the patches and a cross-entropy loss function. After approximately 450 epochs (corresponding to training on approx. 7 × 10^4^ individual patches), the training loss began to plateau (Supplemental Fig. 2). The loss had plateaued by epoch 825, so we added back the held-out cross-validation data and trained the net for approximately 1000 epochs to maximize accuracy on the entire training dataset.

### Step 5: Testing the neural network

Following training, all parameters and weights in the neural network were fixed. Full sized images were classified and the predictions were stored in a text file for analysis. The test sets were held out during training and were only evaluated after the network had been trained.

### Code availability

We used custom python and R scripts, which are provided in the supplementary materials.

## Electronic supplementary material

Supplementary Material

## Data Availability

The nuclear segmentations that were used to train the neural network are freely available under the Creative Commons CC-BY 4.0 license as MultiCellDS digital snapshots^17^ and are available upon request. In addition, the raw H&E images used to generate cell segmentations are available from the website of Biomax.us (IDC, http://www.biomax.us/tissue-arrays/Breast/HBre-Duc140Sur-01).

## Electronic supplementary material

**Supplementary information** accompanies the paper on the *npj Breast Cancer* website (10.1038/s41523-018-0084-4).
