# Supplementary material for: Correlating nuclear morphometric patterns with estrogen receptor status in breast cancer pathologic specimens
Source: NPJ Breast Cancer. 2018 Sep 4;4:32. doi: 10.1038/s41523-018-0084-4 (PMC6123433; doi:10.1038/s41523-018-0084-4)
Supplement: Supplementary file 1 — Supplementary Material [file 41523_2018_84_MOESM1_ESM.pdf]

Supplementary Figures

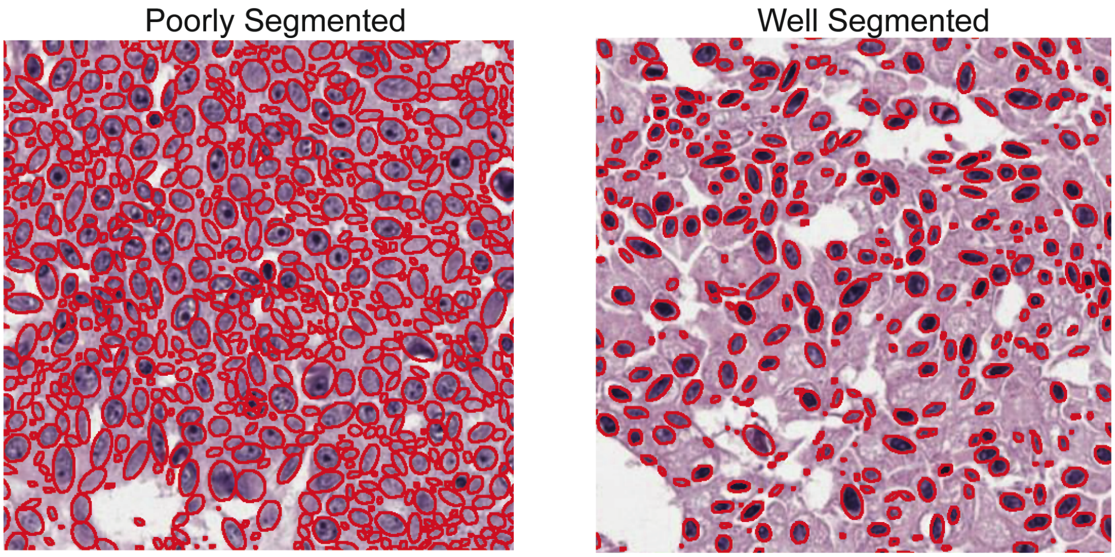

Figure S1. Exemplary over-segmented and well-segmented images. An image segmentation was deemed “well segmented” if it appeared to be more than 70% concordant.

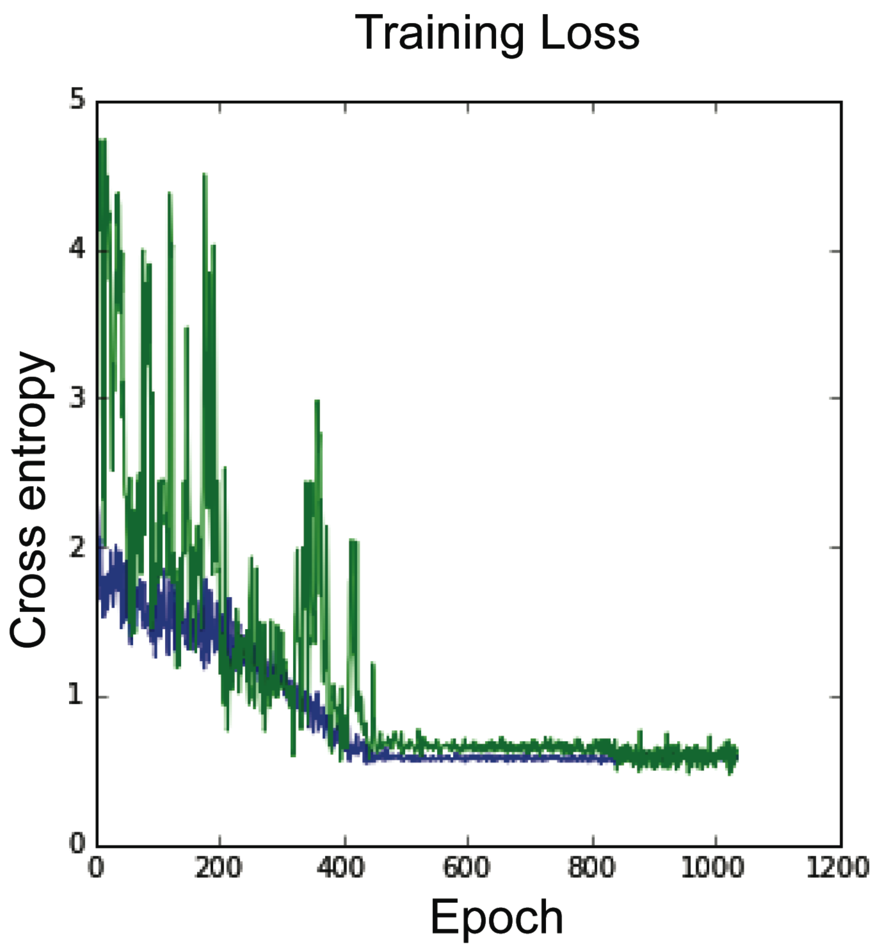

Figure S2. The neural network was trained using adadelta gradient descent with binary cross entropy loss. Initially, 20% of the training data were held-out for cross-validation during the training process (green) and 80% of the training data were used for training (blue). By epoch 825, the loss on the cross-validation data appeared to have converged. At this point, all training data were used to train and validate the model (green) for 200 additional epochs.

Supplementary Code

DESCRIPTION

Submission Code. This contains all the code necessary to obtain the results in the paper titled: "Correlating nuclear morphometric patterns with estrogen receptor status" by Rawat et. al.

Imports

In [ ]:

```
import os
os.environ["CUDA_VISIBLE_DEVICES"]="1"
filelist = []
```

In [2]:

```
def frange(x):
    return range(len(x))

def arrayinarray(arr, InArray):
    out = np.zeros(len(arr), bool)
    for i in frange(arr):
        if arr[i] in InArray:
            out[i] = 1
    return out
```

In [3]:

```
from IPython.display import clear_output
import os, sys, urllib, gzip
```

In [4]:

```
import pickle
sys.setrecursionlimit(10000)

GPU = 1

os.environ["THEANO_FLAGS"]='device=gpu0,mode=FAST_RUN,floatX=float32,nvc
c.flags=-D_FORCE_INLINES'
```

In [5]:

```
from theano import function, config, shared, sandbox
import theano.tensor as T
import numpy
import time
import matplotlib
matplotlib.use('Agg') # Change matplotlib backend, in case we have no X
 server running..
import matplotlib.pyplot as plt

import numpy as np
from IPython.display import Image as IPImage
from PIL import Image
from scipy.ndimage import imread
import scipy
```

In [6]:
```
import theano
print(theano.version.full_version)

import lasagne
print(lasagne.__version__)

from lasagne.layers import get_output, InputLayer, DenseLayer, Upscale2D
Layer, ReshapeLayer, DropoutLayer
from lasagne.nonlinearities import rectify, leaky_rectify, tanh
from lasagne.updates import nesterov_momentum
from lasagne.objectives import categorical_crossentropy
from nolearn.lasagne import NeuralNet, BatchIterator, PrintLayerInfo

from lasagne.layers import Conv2DLayer as Conv2DLayerSlow
from lasagne.layers import MaxPool2DLayer as MaxPool2DLayerSlow
try:
    from lasagne.layers.cuda_convnet import Conv2DCCLayer as Conv2DLayer
Fast
    from lasagne.layers.cuda_convnet import MaxPool2DCCLayer as MaxPool2
DLayerFast
    print('Using cuda_convnet (faster)')
except ImportError:
    from lasagne.layers import Conv2DLayer as Conv2DLayerFast
    from lasagne.layers import MaxPool2DLayer as MaxPool2DLayerFast
    print('Using lasagne.layers (slower)')

def SetDisplaySize(x,y):
    plt.rcParams['figure.figsize'] = (x, y)

def GPUtest():
    ## Segnmentation Net
    vlen = 10 * 1 * 768  # 10 x #cores x # threads per core
    iters = 1000
    rng = numpy.random.RandomState(22)
    x = shared(numpy.asarray(rng.rand(vlen), config.floatX))
    f = function([], T.exp(x))
    print(f.maker.fgraph.toposort())
    t0 = time.time()
    for i in range(iters):
        r = f()
    t1 = time.time()
    print("Looping %d times took %f seconds" % (iters, t1 - t0))
    print("Result is %s" % (r,))
    if numpy.any([isinstance(x.op, T.Elemwise) for x in f.maker.fgraph.t
oposort()]):
        print('Used the cpu')
    else:
        print('Used the gpu')
```

```
0.9.0.dev-RELEASE
0.2.dev1
Using lasagne.layers (slower)
```

In [7]:
```
GPUtest()
```

```
[GpuElemwise{exp,no_inplace}(<CudaNdarrayType(float32, vector)>), HostF
romGpu(GpuElemwise{exp,no_inplace}.0)]
Looping 1000 times took 0.059421 seconds
Result is [ 1.23178029  1.61879349  1.52278066 ...,  2.34847188  2.3326
757   1.0776093 ]
Used the gpu
```

In [8]:
```
import sys


from __future__ import print_function
from matplotlib import pyplot as plt
%matplotlib inline

from PIL import Image
import numpy as np
from skimage.transform import rotate
import numpy as np
import pandas
```

In [9]:
```
from nolearn.lasagne.base import BatchIterator
alpha = theano.shared(np.array(.001, "float32"))
```

In [10]:
```
from matplotlib.mlab import csv2rec
import lasagne.layers as layers
import lasagne as L


import  Misc2
from Misc2 import quickswap
from Misc2 import quickswap as qs

from sklearn.metrics import roc_auc_score
from sklearn.metrics import roc_auc_score

import Misc5
reload(Misc5)

from sklearn.metrics import roc_curve, auc

from sklearn.linear_model import LogisticRegression
from sklearn.discriminant_analysis import QuadraticDiscriminantAnalysis

from sklearn.decomposition import PCA

from nolearn.lasagne.base import BatchIterator

from sklearn.neighbors import KNeighborsClassifier

from sklearn.cluster import KMeans

from nolearn.lasagne.visualize import *
```

In [11]:
```
filelist+=["Misc2", "Misc5"]
```

In [12]:
```
def shapep1(something):
    return np.array(something.reshape((1,) + something.shape),"float32")

def combinebycase(arr, caseid, fxn=np.max):
    # caseid msut be same len as array

    if len(arr) != len(caseid): raise()
    out = []
    for c in np.unique(caseid):
        vals = arr[caseid == c]
        out.append(fxn(vals))
    return np.array(out)
```

Load Data

In the original implementation of this pipeline, we split the Biomax patients into 2 groups (discovery n=57/validation n=56). We also included a second validation set from a different source (Dong et. al. 2016), which contains images from DCIS. While our analysis showed that the neural net trained on the Biomax discovery set significantly predicted ER in both validation sets, we decided to focus on invasive ductal carcinoma in the paper. Hence, even though the code mentions the Dong dataset, we do not report any of these findings.

In [13]:
```
metaBM140 = pandas.read_excel("Meta_BM140.xls")
metaDong = pandas.read_excel("Meta_Dong.xls")
```

In [14]:
```
rawDONG = pickle.load(open("DONG_processed_910.DATA", "r"))
rawBM140 = pickle.load(open("bm140_processed_910.DATA", "r"))

len(rawBM140[1]), len(rawDONG[1])
```

Out[14]:
```
(140, 327)
```

In [15]:
```
fnames = {}
fnames["bm140"] = []
fnames["dong"] = []
```

In [16]:
```
for name in metaBM140["name"].values:
    fnames["bm140"].append("./images/" + name + "_40x.png")
```

In [17]:
```
for name in metaDong["name"].values:
    fnames["dong"].append("./images/" + name + ".tif")
```

In [18]:
```
[(i, feature) for i, feature in enumerate(rawDONG[0])]
```

Out[18]:
```
[(0, 'row'),
 (1, 'col'),
 (2, 'width'),
 (3, 'height'),
 (4, 'angle_0'),
 (5, 'angle_1'),
 (6, 'angle_2'),
 (7, 'angle_3'),
 (8, 'angle_4'),
 (9, 'angle_5'),
 (10, 'maj_minor_ratio'),
 (11, 'area'),
 (12, 'peri'),
 (13, 'circ'),
 (14, 'gray_inten'),
 (15, 'gray_sd')]
```

In [19]:
```
def Construct_Channels(rawData_item, scale = .25):
    #if scale != .1:
    #    print ("original scale is: 0.25 um per pixel. New scale is: %.1
f um per pixel" % (.25 / scale))
    r = np.array(rawData_item[:,0] * scale, int)
    c = np.array(rawData_item[:,1] * scale, int)

    OUT = np.zeros((np.max(r)+1,
                   np.max(c)+1,
                   rawData_item.shape[1] - 2 -2
                    ))
    for i in range(len(r)):
        ## threshold: area > 100 is acceptable
        if rawData_item[i, -5] > 100:
            OUT[r[i], c[i]] = rawData_item[i, 2:-2]
    return OUT

CHANNELS_Dong = np.array([Construct_Channels(d) for d in rawDONG[1]])
CHANNELS_BM140 = np.array([Construct_Channels(d) for d in rawBM140[1]])
```

In [20]:
```
np.sum([np.sum(a[:,:,-5] > 0) for a in CHANNELS_Dong])
```

Out[20]:
```
17767
```

In [21]:
```
from scipy.misc import imresize
```

In [22]:
```
CHANNELS_BM140[0].shape
```

Out[22]:
```
(486, 505, 12)
```

In [23]:
```
SetDisplaySize(10,10)
rgb = imread(fnames["bm140"][0])
plt.imshow(imresize(CHANNELS_BM140[0][:,:,0]>0, (rgb.shape[0:2])))
plt.imshow(rgb, alpha=.25)
plt.savefig("overlay_nuclei_HE.png")
plt.clf()
clear_output()
```

```
<matplotlib.figure.Figure at 0x7f87220d2d90>
```

In [24]:
```
import RRutils.utils
```

In [25]:
```
patches_seg = RRutils.utils.makespacedsquares((CHANNELS_BM140[0:1], np.o
nes(1)), 32, 4)[0]

patches_rgb = RRutils.utils.makespacedsquares(([rgb], np.ones(1)), 32*4*
2, 4)[0]
```

In [26]:
```
SetDisplaySize(4,2)
plt.subplot(1,2,1).imshow(patches_seg[3][:,:,0]>0)
plt.subplot(1,2,2).imshow(patches_rgb[3])
```

Out[26]:
```
<matplotlib.image.AxesImage at 0x7f84f0bb5710>
```

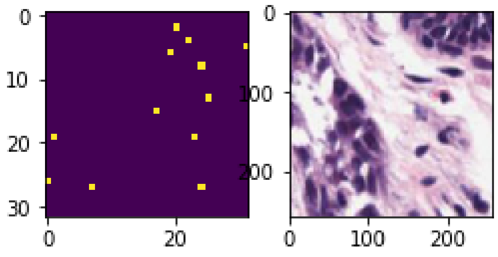

Filter out small/poorly segmented images/ images lacking ground truth

In [27]:
```
## Dong Test Set - DCIS
Dong_valid = (metaDong.has_er_meas.values == True) & (metaDong.seg_ok.va
lues == True) & (metaDong.size_gt_52 == True)
X_Dong_er_test = CHANNELS_Dong[ Dong_valid ]
Y_Dong_er_test = metaDong.ERBOOL.values[ Dong_valid ]
PID_Dong_er_test = metaDong.PID.values[Dong_valid ]
name_Dong_er_test = metaDong.name.values[Dong_valid ]


print(np.unique(PID_Dong_er_test).shape)
```

```
(71,)
```

In [28]:
```
## BM ER Set (TOTAL DATASET)
X_BM_ER = CHANNELS_BM140[ metaBM140.Keep_ER.values]
Y_BM_ER = metaBM140.ERBOOL.values[ metaBM140.Keep_ER.values]
PID_BM_ER = metaBM140.PID.values[ metaBM140.Keep_ER.values]
name_BM_ER = metaBM140.name.values[ metaBM140.Keep_ER.values]


print(np.unique(PID_BM_ER).shape, "total patients in ER+/- set" )
print(X_BM_ER.shape, Y_BM_ER.shape, PID_BM_ER.shape)
```

```
(113,) total patients in ER+/- set
(113,) (113,) (113,)
```

In [29]:
```
bm_er_fnames = metaBM140.name[metaBM140.Keep_ER.values == 1].values
```

In [30]:
```
## make a training/test BM set
```

In [31]:
```
np.random.seed(42)
ran = np.random.permutation(113)

ntrain = 57

X_BM_ER_train = X_BM_ER[ran][:ntrain]
Y_BM_ER_train = Y_BM_ER[ran][:ntrain]
PID_BM_ER_train = PID_BM_ER[ran][:ntrain]
name_BM_ER_train = name_BM_ER[ran][:ntrain]

X_BM_ER_test = X_BM_ER[ran][ntrain:]
Y_BM_ER_test = Y_BM_ER[ran][ntrain:]
PID_BM_ER_test = PID_BM_ER[ran][ntrain:]
name_BM_ER_test = name_BM_ER[ran][ntrain:]
```

In [32]:
```
len(bm_er_fnames)
```

Out[32]:
```
113
```

In [33]:
```
bm_er_fnames[ran][:ntrain]
```

Out[33]:
```
array([u'H02', u'A05', u'D06', u'F12', u'A13', u'D13', u'F13', u'F09',
       u'E01', u'A14', u'I12', u'C14', u'H09', u'J13', u'B09', u'A01',
       u'G01', u'C04', u'H06', u'E09', u'J05', u'I04', u'B02', u'C09',
       u'F10', u'J12', u'J09', u'B13', u'D11', u'J10', u'G13', u'B05',
       u'C08', u'J04', u'A12', u'C11', u'F04', u'F08', u'C06', u'D08',
       u'A06', u'E11', u'F07', u'D03', u'C13', u'B06', u'I14', u'C12',
       u'E12', u'A09', u'D10', u'F11', u'H11', u'C05', u'B10', u'I03',
       u'I08'], dtype=object)
```

In [34]:
```
bm_er_fnames[ran][ntrain:]
```

Out[34]:
```
array([u'C03', u'A11', u'H10', u'E04', u'B03', u'G14', u'C02', u'A04',
       u'B07', u'D02', u'I06', u'A07', u'G02', u'I11', u'I01', u'E10',
       u'E05', u'G05', u'D14', u'G08', u'F03', u'J11', u'H14', u'D07',
       u'E14', u'E02', u'I09', u'E13', u'G04', u'C10', u'J06', u'F01',
       u'F05', u'I07', u'D01', u'C07', u'I13', u'A02', u'E08', u'B12',
       u'A03', u'C01', u'H13', u'J01', u'G03', u'H12', u'H07', u'J14',
       u'B11', u'F02', u'F14', u'J07', u'B04', u'I05', u'E06', u'J03'],
dtype=object)
```

In [35]:
```
for item in (Y_BM_ER_train, X_BM_ER_train, Y_BM_ER_test, X_BM_ER_test):
    print (item.shape)
    try: print(np.mean(item))
    except: pass
```

```
(57,)
0.701754385965
(57,)
(56,)
0.732142857143
(56,)
```

In [36]:
```
[(i, feature) for i, feature in enumerate(rawDONG[0][2:-2])]
```

Out[36]:
```
[(0, 'width'),
 (1, 'height'),
 (2, 'angle_0'),
 (3, 'angle_1'),
 (4, 'angle_2'),
 (5, 'angle_3'),
 (6, 'angle_4'),
 (7, 'angle_5'),
 (8, 'maj_minor_ratio'),
 (9, 'area'),
 (10, 'peri'),
 (11, 'circ')]
```

Display the 12 D vector

In [37]:
```
plt.gray()
SetDisplaySize(20,6)
plt.figure(facecolor='white')

for i, feature in enumerate(rawDONG[0]):
    if i in [0,1,14,15]:
        continue
    else:
        i -= 2

        sp = plt.subplot(2,6,i+1)
        sp.imshow(X_BM_ER_train[4][300:364,600:664,i] *10, cmap=plt.cm.g
ray )

        if sp == plt:
            plt.title("%s" % (feature))
            plt.gca().xaxis.set_major_locator(plt.NullLocator())
            plt.gca().yaxis.set_major_locator(plt.NullLocator())
        else:
            sp.set_title( "%s" % (feature) )
            sp.xaxis.set_major_locator(plt.NullLocator())
            sp.yaxis.set_major_locator(plt.NullLocator())

        #plt.show()
#plt.savefig("Illustration_of_12D_vector.png")
#plt.clf()
plt.show()        #
```

```
<matplotlib.figure.Figure at 0x7f8480f1e490>
```

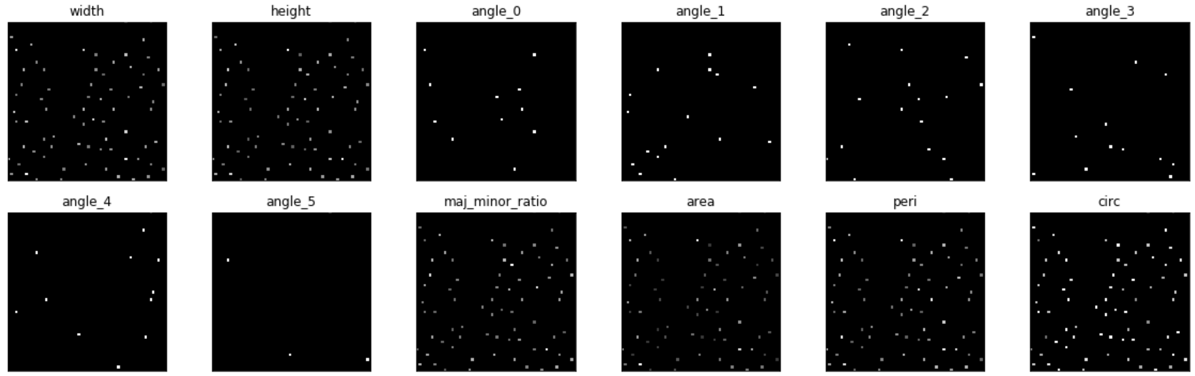

define model

In [38]:
```
from lasagne.layers import ConcatLayer

np.random.seed(42)
nF = 32
nChan = 12
imagesize = None #401
Conv2DLayer = Conv2DLayerFast
nL = lasagne.nonlinearities.leaky_rectify
stride = theano.shared(np.array(1, int))
dropP = .5
nfilters = 8

CONTROL = 0
ROUND = 10
in1 = lasagne.layers.input.InputLayer(**{'name': 'base', 'shape': (None,
 nChan, imagesize,imagesize) }) ## assume 41x41

if 1:
    padding = 20
    feature1 = []
    out1 = lasagne.layers.Pool2DLayer(in1, name="pool_0", mode= 'average
_exc_pad', stride=(1,1), pool_size=5)  # size 37

    f1 = in1
    for q in range(5):
        f1 = Conv2DLayer(**{'name':'C%.2i'%q, 'incoming':f1, 'filter_siz
e':3, 'num_filters': 16 * 2**q , 'pad':'same', 'nonlinearity':nL})
        f1 = MaxPool2DLayerFast(f1, pool_size=(2,2))


NFCNs = 512/2

out1 = Conv2DLayer(**{'incoming':f1, 'filter_size': 1, 'num_filters': NF
CNs, 'pad':'same', 'nonlinearity':nL, 'name':'FC-1'})
out1 = DropoutLayer(out1, p=.5)
out1 = DropoutLayer(out1, p=.5)
out1 = Conv2DLayer(**{'incoming':out1, 'filter_size': 1, 'num_filters':
1, 'pad':'same', 'nonlinearity':nL, 'name':'preds'})


GM_layer = lasagne.layers.GlobalPoolLayer(out1, pool_function=T.mean, na
me="globalmean_prob")
clf_layer = lasagne.layers.BatchNormLayer(GM_layer)
clf_layer = lasagne.layers.FlattenLayer(clf_layer)
```

In [39]:
```
from lasagne.layers import Layer
def bce_sigmoid(pred, targ):
    pred = T.clip(pred, .001, .999)
    targ = T.clip(targ, .001, .999)
    return lasagne.objectives.binary_crossentropy(pred, targ)
```

initialize

In [40]:
```
np.random.seed(42)


eval_size = .2

net_trip = NeuralNet(

    layers=clf_layer,
    max_epochs=1,
    batch_iterator_train=BatchIterator(1, shuffle=True), # shuffle on my
 own
    update= lasagne.updates.adadelta,
    regression=True, # dont use crossentropy
    verbose=1,

    objective_loss_function = bce_sigmoid,

    eval_size=eval_size,
    objective_l2 = .00001,
    )


net_trip.initialize()
PrintLayerInfo()(net_trip)
```

```
# Neural Network with 459955 learnable parameters

## Layer information

  #  name             size
---  ---------------  -------------
  0  base             12xNonexNone
  1  C00              16xNonexNone
  2                   16xNonexNone
  3  C01              32xNonexNone
  4                   32xNonexNone
  5  C02              64xNonexNone
  6                   64xNonexNone
  7  C03              128xNonexNone
  8                   128xNonexNone
  9  C04              256xNonexNone
 10                   256xNonexNone
 11  FC-1             256xNonexNone
 12                   256xNonexNone
 13                   256xNonexNone
 14  preds            1xNonexNone
 15  globalmean_prob  1
 16                   1
 17                   1
```

train the net

In [41]:
```
netname = "net_trip" # "net_trip"
nettouse = eval(netname)
```

In [42]:
```
nettouse.load_params_from("final_net_params_fulltrain.params")
```

```
Loaded parameters to layer 'C00' (shape 16x12x3x3).
Loaded parameters to layer 'C00' (shape 16).
Loaded parameters to layer 'C01' (shape 32x16x3x3).
Loaded parameters to layer 'C01' (shape 32).
Loaded parameters to layer 'C02' (shape 64x32x3x3).
Loaded parameters to layer 'C02' (shape 64).
Loaded parameters to layer 'C03' (shape 128x64x3x3).
Loaded parameters to layer 'C03' (shape 128).
Loaded parameters to layer 'C04' (shape 256x128x3x3).
Loaded parameters to layer 'C04' (shape 256).
Loaded parameters to layer 'FC-1' (shape 256x256x1x1).
Loaded parameters to layer 'FC-1' (shape 256).
Loaded parameters to layer 'preds' (shape 1x256x1x1).
Loaded parameters to layer 'preds' (shape 1).
Loaded parameters to layer 'batchnorm16' (shape 1).
Loaded parameters to layer 'batchnorm16' (shape 1).
Loaded parameters to layer 'batchnorm16' (shape 1).
Loaded parameters to layer 'batchnorm16' (shape 1).
```

In [43]:
```
raise()  ## comment to train


SetDisplaySize(5,5)
#np.random.seed(42)

net_trip_mean = net_trip

paramfilename = '918%s.Round%i.params.3' % (netname, ROUND)
histfilename = '918%s.Round%i.hist' % (netname, ROUND)
if VERSION == -1:
    FOLD = 2
    batchsize = 228
    net_trip_mean.batch_iterator_train.batch_size = batchsize
    net_trip_mean.max_epochs = 1



    for BigEpoch in range(100): #30000

        alpha.set_value(100.1)

        if BigEpoch % 1 == 0:
            clear_output()

            trainloss = [item["train_loss"] for item in nettouse.train_h
istory_]
            validloss = [item["valid_loss"] for item in nettouse.train_h
istory_]

            plt.plot(trainloss[0:])
            plt.plot(validloss[0:])

            #plt.xlim(2000, 16000)
            #plt.ylim(0,.7)

            plt.show()
            nettouse.save_params_to(paramfilename)

            if 1:
                f = open(histfilename, "w")
                trainhist = str(net_trip_mean.train_history_)
                f.write(trainhist)
                f.close()
                pass

        size = 40

        x,y = Misc2.genData_boolean([X_BM_ER_train, Y_BM_ER_train], size
=100, fold=5)

        if 1:
            ## after 800 epochs
            ran1 = np.random.permutation(len(x))

            nettouse.fit(qs(x[ran1]), np.float32(y[ran1] == 0) )
```

```
        else:
            #print(net_trip.layers_[-1].b.get_value(), net_trip.layers_
[-1].W.get_value())
            #print(net_trip.layers_[-1].b.get_value(), net_trip.layers_
[-1].W.get_value())
            nettouse.fit(qs(x), np.float32(y==0) )

    print("done")
```

```
TypeErrorTraceback (most recent call last)
<ipython-input-43-746a72defc69> in <module>()
----> 1 raise()  ## comment to train
      2
      3
      4 SetDisplaySize(5,5)
      5 #np.random.seed(42)

TypeError: exceptions must be old-style classes or derived from BaseExc
eption, not tuple
```

Assess the model's performance on "training" data

In [46]:

```
phat1 = []
F1_1 = []

for i in frange(X_BM_ER_train):

    inp = shapep1(X_BM_ER_train[i])
    #print(inp.shape)
    p = nettouse.predict_proba(qs(inp))
    phat1.append(p)

    f = net_trip.predict_proba(qs(inp))
    F1_1.append(f)

phat1 = np.array(phat1).flatten()
1 - roc_auc_score(Y_BM_ER_train, phat1.flatten())
```

Out[46]: 0.70147058823529407

In [47]:
```
SetDisplaySize(3,3)
plt.scatter(Y_BM_ER_train, np.array(phat1).flatten(), color="black")
```

Out[47]: <matplotlib.collections.PathCollection at 0x7f84d9f4f750>

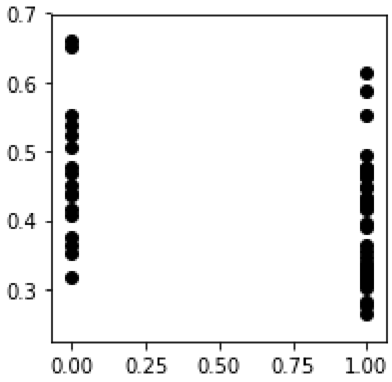

In [48]:
```
import mahotas as mh
```

Define some functions for visualization

In [49]:

```
from skimage import color


def overlay2(big,small, thresh=.5 * 255,  showmask = False, xmax=2500, y
max=3000):

    if 0:
        pass
    else:

        scaled = scipy.misc.imresize(small, (big.shape[0], big.shape[1
]), interp="bicubic")
        scaled = scaled * small.max()
        scaled[0,0] = 0
        scaled[-1,-1] = 255
        print(small.max(), "smallmax", scaled.max())

        if showmask:
            plt.imshow(scaled)
            plt.show()

        print(scaled.shape)
        scaled = scaled[:min(big.shape[0], scaled.shape[0]), :min(big.sh
ape[1], scaled.shape[1])]
        rgbout = big[:min(big.shape[0], scaled.shape[0]), :min(big.shape
[1], scaled.shape[1]), :3]

        print(scaled.shape, rgbout.shape)
        grayout = color.gray2rgb(color.rgb2gray(rgbout)) * 255

        print(grayout.shape, scaled.shape)
        grayout = mh.overlay(grayout[:,:,0], scaled > thresh)

        #grayout[scaled>thresh] = rgbout[scaled>thresh]

        if (xmax and ymax):
            plt.imshow(np.array(grayout, "uint8")[:xmax,:ymax])
        else:
            plt.imshow(np.array(grayout, "uint8"))

        #plt.hold(True)
        #plt.imshow(scaled, alpha=.5)
        #plt.show()
        return


def overlay3(big,small, thresh=.5 * 255, showmask = False):
    """Alpha channel"""

    print(big.shape)
    print(small.shape)
    scaled = scipy.misc.imresize(small, (big.shape[0], big.shape[1]), in
terp="bicubic")  #> thresh
    scaled = np.uint8(scaled * small.max())

    scaled[0,0] = 0
```

```
    scaled[-1,-1] = 255
    #scaled = (1.00001 * scaled/scaled.max()) * 255

    if showmask:
        plt.imshow(scaled)
        plt.show()

    print(scaled.shape)
    scaled = scaled[:min(big.shape[0], scaled.shape[0]), :min(big.shape[
1], scaled.shape[1])]
    rgbout = big[:min(big.shape[0], scaled.shape[0]), :min(big.shape[1],
 scaled.shape[1]), :3]

    print(scaled.shape, rgbout.shape)
    grayout = color.rgb2gray(rgbout)

    #grayout[scaled>threshold] = rgbout[scaled>threshold]

    alphad = np.dstack((rgbout, scaled))

    #out = grayout * (1 - np.dstack((scaled, scaled, scaled))) +  rgbout
 * ( np.dstack((scaled, scaled, scaled)))

    plt.imshow(grayout)
    plt.hold(True)

    plt.imshow(alphad, alpha=.9)

    #plt.show()
    #return np.array(out, "uint8")
```

In [50]:

```
import pandas as pd

def Find_Optimal_Cutoff(target, predicted):
    """ Find the optimal probability cutoff point for a classification m
odel related to event rate
    Parameters
    ----------
    target : Matrix with dependent or target data, where rows are observ
ations

    predicted : Matrix with predicted data, where rows are observations

    Returns
    -------
    list type, with optimal cutoff value

    """
    fpr, tpr, threshold = roc_curve(target, predicted)
    i = np.arange(len(tpr))
    roc = pd.DataFrame({'tf' : pd.Series(tpr-(1-fpr), index=i), 'thresho
ld' : pd.Series(threshold, index=i)})
    roc_t = roc.ix[(roc.tf-0).abs().argsort()[:1]]

    return list(roc_t['threshold'])[0]
```

In [51]:
```
Find_Optimal_Cutoff(Y_BM_ER_train, phat1.flatten())
```

Out[51]: 0.43557891

Test the Trained CLF on the (held-out) Biomax Test Set

These data were held out during training

In [52]:
```
phat2 = []

for i in frange(X_BM_ER_test):

    inp = shapep1(X_BM_ER_test[i])
    #print(inp.shape)
    p = nettouse.predict_proba(qs(inp))
    phat2.append(p)

phat2 = np.array(phat2).flatten()
1 - roc_auc_score(Y_BM_ER_test, phat2.flatten())
```

Out[52]: 0.71869918699186996

In [53]:
```
SetDisplaySize(3,3)

a = plt.scatter(Y_BM_ER_test, phat2.flatten(), color="black")
```

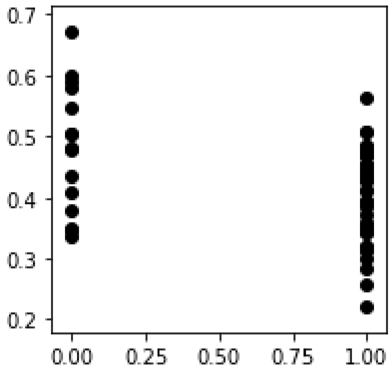

Evaluate model on (held-out) DONG Dataset

Also held out during training

In [54]:
```python
predmaps_3 = []

phat3 = []
donger = X_Dong_er_test
dongerY = Y_Dong_er_test
for i in frange(donger):

    inp = shapep1(donger[i])
    p = nettouse.predict_proba(qs(inp))
    phat3.append(p)

phat3 = np.array(phat3).flatten()
1 - roc_auc_score(dongerY, phat3)
```

Out[54]: 0.81492785793562705

In [55]:
```python
pid3 = PID_Dong_er_test
phat3_cs = combinebycase(phat3, pid3)

dongerY_cs = combinebycase(dongerY, pid3)
```

In [56]:
```python
name_Dong_er_cs = combinebycase(name_Dong_er_test, pid3)
```

In [57]:
```python
len(dongerY_cs), len(np.unique(name_Dong_er_cs))
```

Out[57]: (71, 71)

In [58]:
```python
SetDisplaySize(3,3)
a = plt.scatter(dongerY, phat3, color="black")
```

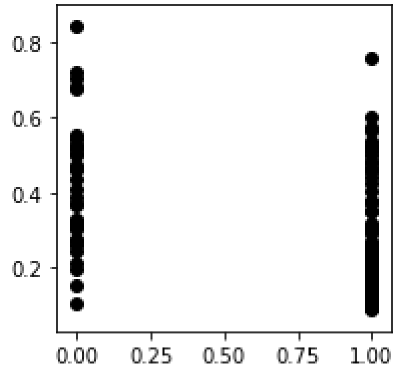

In [59]:
```
SetDisplaySize(3,3)
a = plt.scatter(dongerY_cs, phat3_cs, color="black")
```

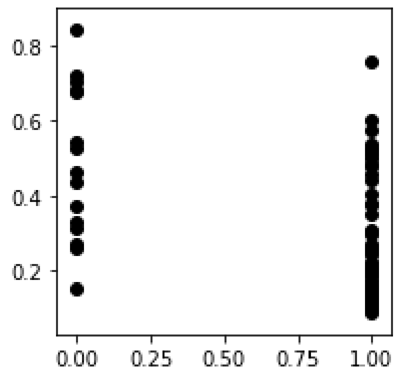

What did it learn?

In [60]:
```
def DensePredictor(image, title = None):
    sqs, nrow, ncol = RRutils.utils.makesubsquares(image, 32, (16,16))
    print (nrow,ncol)



    preds = net_trip.predict(np.array(quickswap(np.array(sqs)), "float3
2"))

    plt.imshow(preds.reshape(nrow, ncol) , interpolation="bicubic")
```

In [61]:
```
DensePredictor(X_BM_ER_test[3])
```

31 52

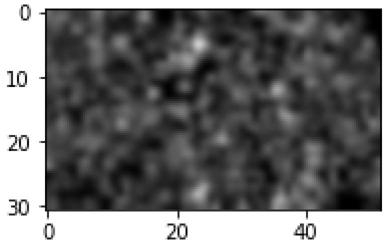

In [62]:
```
from Misc2 import *
def genDatatandem(dataset, size=200, channels=3, fold=1, introtate=0):
    sampleFold = 1 + introtate * 4

    imageList = dataset[0] #list of images
    rgbimages = dataset[2]


    labels = [] #list of labels

    out = [] #output list of images
    rgbout = []

    #for fold_n in range(fold):
    for i in range(len(imageList)):

        dat2 = imageList[i]
        rgbresized = scipy.misc.imresize(rgbimages[i], (dat2.shape[0], d
at2.shape[1]) )

        #print(rgbresized.shape)
        for fold_n in range(fold):
            myseed = np.random.randint(0,100000)
            #print(myseed)
            label = dataset[1][i]
            if ((dat2.shape[0] < size) or (dat2.shape[1] < size)):
                continue

            np.random.seed(myseed)
            out += [np.array(flipAndRandom(dat2, size), 'float32') for _
i in range(sampleFold)]   ###################

            np.random.seed(myseed)
            rgbout += [np.array(flipAndRandom(rgbresized, size), 'float3
2') for _i in range(sampleFold)]

            labels += [label for _i in range(sampleFold)]

    out = np.array(out)
    #out = resizeX(out, shrinkfactor)
    return out, np.array(labels), rgbout
```

In [63]:
```
bm_er_ims = []

for fname in bm_er_fnames:
    fpath = "./images/"+fname+"_40x.png"

    bm_er_ims.append(  scipy.misc.imresize(np.array(imread(fpath))[:,:,0
:3] , .25 )  )
```

In [64]:
```
bm_er_ims = np.array(bm_er_ims)
```

In [69]:
```
np.random.seed(42)
samples, sample_gt, rgbsamples = genDatatandem([X_BM_ER, Y_BM_ER, bm_er_
ims], size=64, fold=100)

keep = np.array([s[:,:,0].sum() > 0 for s in samples])
samples = samples[keep == 1]
rgbsamples = np.uint8(rgbsamples)[keep==1]
sample_gt = sample_gt[keep == 1]
```

In [70]:
```
sample_preds = net_trip.predict(qs(samples)).flatten()
```

In [71]:
```
SetDisplaySize(3,3)
print(sample_preds.shape, sample_preds.min(), sample_preds.max())
plt.hist(sample_preds)
```

```
(11161,) 0.0891686 1.07333
```

Out[71]:
```
(array([  811.,  2206.,  3326.,  2520.,  1364.,   608.,   226.,    67.,
          27.,     6.]),
 array([ 0.08916862,  0.18758512,  0.28600162,  0.38441812,  0.4828346
2,
         0.58125112,  0.67966762,  0.77808412,  0.87650062,  0.9749171
2,
         1.07333362]),
 <a list of 10 Patch objects>)
```

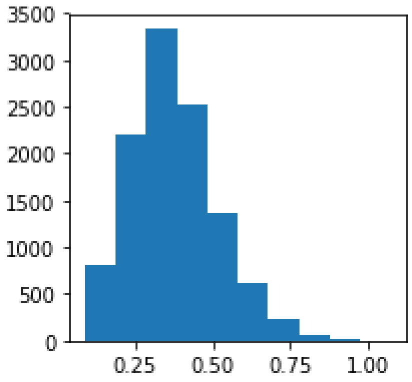

In [72]:
```
sample_preds
```

Out[72]:
```
array([ 0.43866351,  0.22833519,  0.36717215, ...,  0.50394499,
        0.21490578,  0.35884726], dtype=float32)
```

In [73]:
```
order = np.argsort(sample_preds)
```

In [74]:
```
## from low to high probability of ER
samples_ordered = samples[order]
sample_preds_ordered = sample_preds[order]

rgb_samples_ordered = rgbsamples[order]
```

In [75]:
```
np.concatenate((np.ones(6), np.ones(1)))
```
Out[75]:
```
array([ 1.,  1.,  1.,  1.,  1.,  1.,  1.])
```
In [76]:
```
featindx = [0,1,-4,-3,-2,-1]
sixfeatnames = np.array(["width", "height", "mmr", "area", "peri", "cir
c"])
```
In [77]:
```
print("only dealing with first 6 features")

def summarizesample(sample):
    """Summarizes a SINGLE SAMPLE"""
    # for each feature, provide a mean across # of cells, variation, tot
al no of cells
    mask = sample[:,:,0] != 0
    cells = sample[mask]

    ncells = np.sum(mask).reshape(-1)

    return np.hstack(( cells.mean(axis=0)[featindx], cells.var(axis=0)[f
eatindx],  ncells))
```
```
only dealing with first 6 features
```
collect stats for each image in the dataset

first six features = mean of the 6 features,

next six: variance of the 6 feautres IN THE SAMPLE

last feature: n cells in the sample

In [79]:
```
samplestats = np.array([summarizesample(s) for s in samples_ordered])
samplestats.shape
```
Out[79]:
```
(11161, 13)
```
In [80]:
```
samplestats_normed = samplestats / samplestats.max(axis=0)
```
In [81]:
```
## for each bracket get mean and 95% CI: store as list of lists: [ [mea
n, min95, max95], [mean, min95, max95] ..]
```
In [82]:
```
import scikits.bootstrap as bootstrap
```

In [83]:
```
## Calculate MEANS
brackets = 15
intervals = samples_ordered.shape[0]/brackets

bracketmean = []
bracketci_lo = []
bracketci_hi = []



for i in range(brackets):
    bracket = samplestats_normed[i*intervals: (i+1) * intervals]

    m = []
    cilow = []
    cihigh = []

    for j in range(13): ## by feature
        data = bracket[:, j]
        m.append( data.mean() )
        ci = bootstrap.ci(data, np.mean )
        cilow.append(ci[0])
        cihigh.append(ci[1])
    bracketmean.append(m)
    bracketci_lo.append(cilow)
    bracketci_hi.append(cihigh)

## these will have the MEANS of the 13 things: eg. mean_bracket(  mean_s
ample )
## these will be plotted as a line

#brackets are the CI of these features

## 15 rows = 15 quadrants, 13 columns = 13 features
```

In [84]:
```
bracketci_hi = np.array(bracketci_hi)
bracketci_lo = np.array(bracketci_lo)
bracketmean  = np.array(bracketmean)
```

In [85]:
```
## There are 15 brackets adn 13 features at each bracket (e.g. 13 lines
 to plot)
bracketci_hi.shape, bracketmean.shape
```

Out[85]: ((15, 13), (15, 13))

In [87]:
```
sixfeatnames2 = ('width', 'height', 'mmr', 'area', 'perimeter', 'circula
rity')
```

In [88]:
```
SetDisplaySize(10,10)

for featurei in range(6):
    if featurei <6:
        line = plt.plot(np.arange(15), np.array(bracketmean)[:,featurei
], label=sixfeatnames2[featurei] )[0]

        color = line.get_color()

        for x,y in zip(np.arange(15), np.array(bracketmean)[:,featurei
]):
            yerr = .5 * (bracketci_hi[x, featurei] - bracketci_lo[x, fea
turei])
            plt.errorbar(x, y, xerr=0.0, yerr=yerr, c = color)

#legend = plt.legend(loc=(1.1,.5), shadow=True)
plt.yticks(plt.yticks()[0], fontsize=14)
plt.xticks(plt.xticks()[0], fontsize=14)
plt.title("Patch Mean", fontsize=20)
plt.ylabel("normalized value", fontsize=20)
plt.show()


for featurei in range(6, 13):
    if featurei <12:
        line = plt.plot(np.arange(15), np.array(bracketmean)[:,featurei
], label=sixfeatnames2[featurei - 6] )[0]

        color = line.get_color()

        for x,y in zip(np.arange(15), np.array(bracketmean)[:,featurei
]):
            yerr = .5 * (bracketci_hi[x, featurei] - bracketci_lo[x, fea
turei])
            plt.errorbar(x, y, xerr=0.0, yerr=yerr, c = color)

    else:
        plt.plot(np.array(bracketmean)[:,featurei], 'k-', label="cellcou
nt")

        legend = plt.legend(loc=(1.1, .5), shadow=True, fontsize=20)
        plt.title("Patch Variance", fontsize=20)
        #for tick in plt.xticks():
        #       tick.label.set_fontsize(14)
        plt.yticks(plt.yticks()[0], fontsize=14)
        plt.xticks(plt.xticks()[0], fontsize=14)
        plt.show()




## Save predictions
predictions = np.vstack((

    np.vstack((np.array(["bm-train"]*len(phat1)),name_BM_ER_train, Y_BM_
ER_train, phat1)).T,
```

```
np.vstack((np.array(["bm-test"]*len(phat2)),name_BM_ER_test , Y_BM_ER_te
st,phat2)).T,
np.vstack((np.array(["dong-test-image-level"]*len(phat3)),name_Dong_er_t
est, dongerY, phat3)).T,
np.vstack((np.array(["dong-test-patient-level"]*len(phat3_cs)),name_Dong
_er_cs, dongerY_cs, phat3_cs)).T

    ))

np.savetxt("predictions.csv", predictions, fmt="%s", delimiter=",")
```

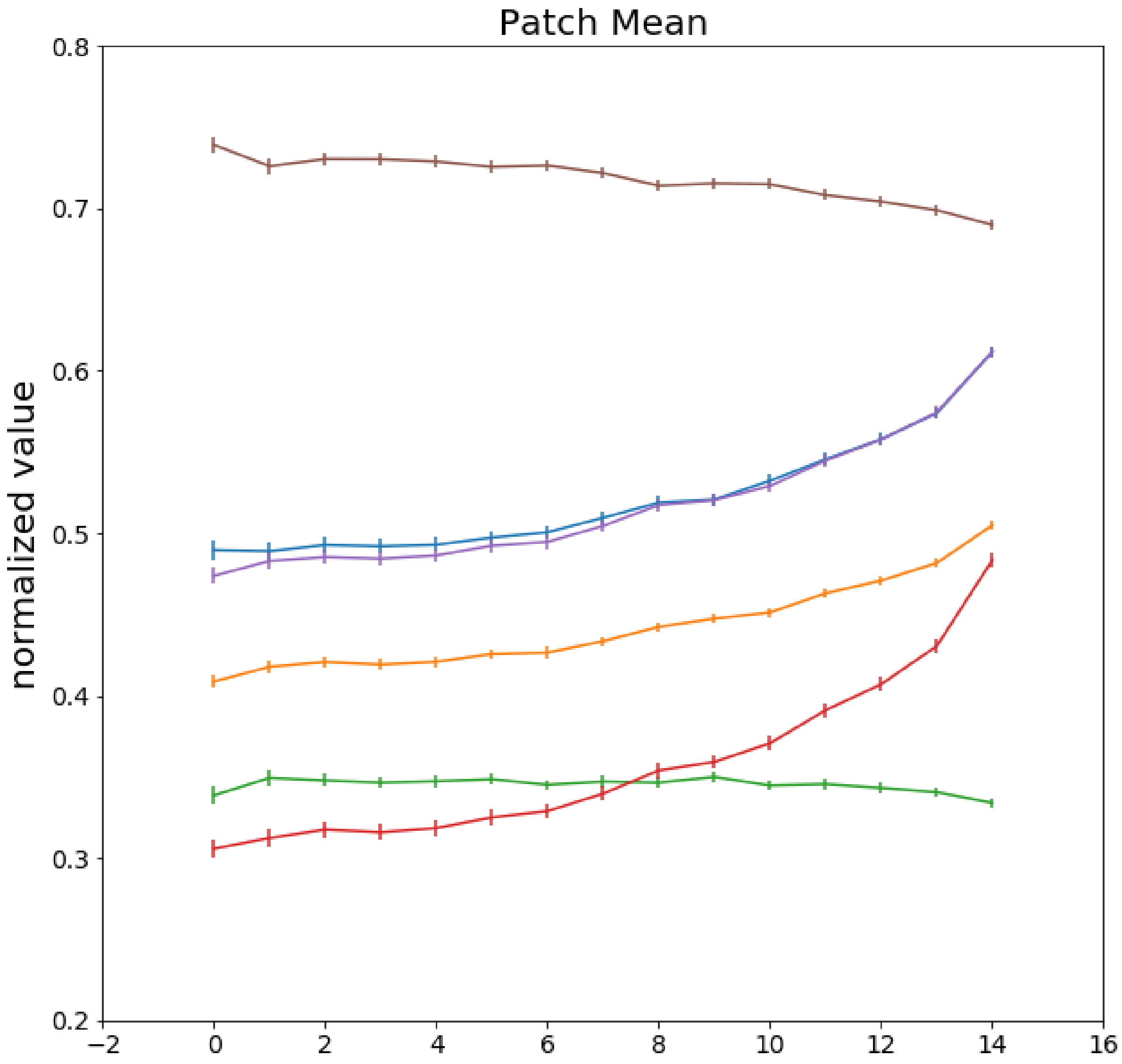
Patch Mean
normalized value
0.8
0.7
0.6
0.5
0.4
0.3
0.2
−2
0
2
4
6
8
10
12
14
16

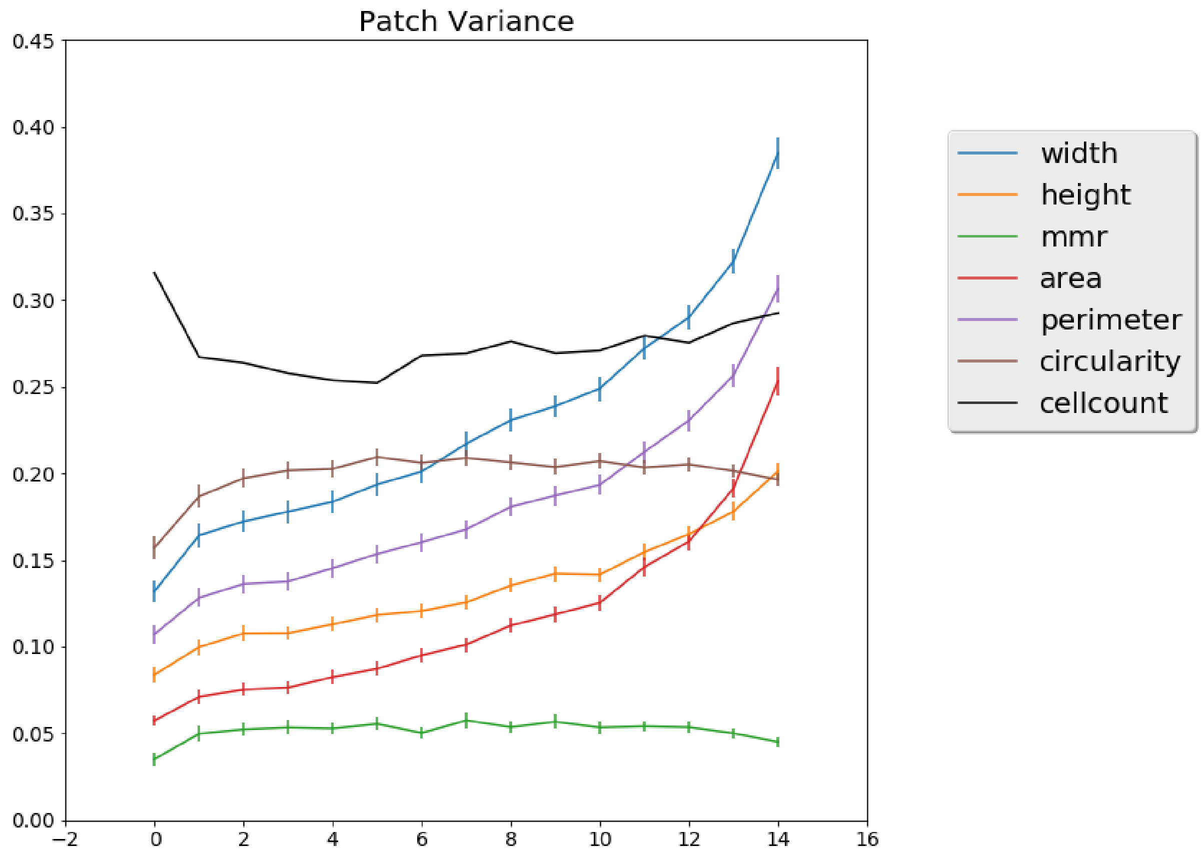

In [ ]:

```
SetDisplaySize(10,10)
plt.figure(facecolor="white")
plot_loss(net_trip)
if 0:
    plt.savefig("trainingloss920.png")
```

```
### this script prints out the CI on the predictions outputted from
the network

preds  = read.csv("predictions.csv",sep = ",")

unique(preds[,1])


library(pROC)

for (set in c("bm-test", "bm-train", "dong-test-image-level", "dong-
test-patient-level")) {

  print(set)

  valid = preds[,1] == set

  gt = preds[,3][valid]
  p  = preds[,4][valid]

  #print(valid)
  print( ci(gt,p) )
  print(auc(gt,p))
}
```

Predictions

Position	Dataset	ImageID	ER_status	Prediction	Grade	Used in study
A1	bm-train	A01	1	0.32406068	2	1
A2	bm-test	A02	1	0.48345652	2	1
A3	bm-test	A03	1	0.50572175	1.5	1
A4	bm-test	A04	1	0.4118703	2	1
A5	bm-train	A05	0	0.41702446	2	1
A6	bm-train	A06	1	0.44889027	1.5	1
A7	bm-test	A07	1	0.37199688	1.5	1
A9	bm-train	A09	1	0.47320604	1.5	1
A11	bm-test	A11	0	0.34952173	2	1
A12	bm-train	A12	1	0.34588736	2	1
A13	bm-train	A13	0	0.43557891	2	1
A14	bm-train	A14	0	0.65930474	1	1
B2	bm-train	B02	1	0.30883408	2	1
B3	bm-test	B03	1	0.43276647	2.5	1
B4	bm-test	B04	0	0.48161587	2	1
B5	bm-train	B05	1	0.43380895	2	1
B6	bm-train	B06	1	0.31727934	2	1
B7	bm-test	B07	0	0.41005701	2	1
B9	bm-train	B09	0	0.37628028	1.5	1
B10	bm-train	B10	0	0.55321419	2	1
B11	bm-test	B11	0	0.34523419	2	1
B12	bm-test	B12	1	0.22134452	1.5	1
B13	bm-train	B13	0	0.40693051	2	1
C1	bm-test	C01	1	0.38599506	2	1
C2	bm-test	C02	1	0.35155219	2	1
C3	bm-test	C03	1	0.34233952	1	1
C4	bm-train	C04	1	0.27590972	1.5	1
C5	bm-train	C05	1	0.3144159	1.5	1
C6	bm-train	C06	1	0.42752177	2	1
C7	bm-test	C07	1	0.39096004	1.5	1
C8	bm-train	C08	1	0.32917869	2	1
C9	bm-train	C09	1	0.3030932	1.5	1
C10	bm-test	C10	0	0.43541142	1.5	1
C11	bm-train	C11	1	0.35646942	2	1
C12	bm-train	C12	0	0.36276385	2	1
C13	bm-train	C13	1	0.47666729	1	1
C14	bm-train	C14	1	0.33144116	1	1
D1	bm-test	D01	1	0.31930378	1	1
D2	bm-test	D02	1	0.45073405	2	1
D3	bm-train	D03	1	0.42285973	2	1

D6	bm-train	D06	1	0.42172593	2	1
D7	bm-test	D07	1	0.29921189	2	1
D8	bm-train	D08	1	0.33410677	1	1
D10	bm-train	D10	0	0.46991271	1	1
D11	bm-train	D11	0	0.50539565	1.5	1
D13	bm-train	D13	1	0.47578496	1.5	1
D14	bm-test	D14	0	0.5890581	1.5	1
E1	bm-train	E01	0	0.451013	1.5	1
E2	bm-test	E02	0	0.50443363	2	1
E4	bm-test	E04	1	0.31623808	2	1
E5	bm-test	E05	1	0.25779769	2	1
E6	bm-test	E06	1	0.2841965	1	1
E8	bm-test	E08	1	0.43871439	2	1
E9	bm-train	E09	1	0.61303037	1.5	1
E10	bm-test	E10	1	0.3141036	2	1
E11	bm-train	E11	1	0.49475098	2	1
E12	bm-train	E12	1	0.33820877	2	1
E13	bm-test	E13	1	0.43857884	2.5	1
E14	bm-test	E14	1	0.4493506	2	1
F1	bm-test	F01	1	0.46988118	2	1
F2	bm-test	F02	1	0.34868679	1.5	1
F3	bm-test	F03	1	0.3209745	1.5	1
F4	bm-train	F04	1	0.33073846	2	1
F5	bm-test	F05	0	0.59927523	2	1
F7	bm-train	F07	1	0.28386176	2	1
F8	bm-train	F08	1	0.26523137	2.5	1
F9	bm-train	F09	1	0.39753431	2	1
F10	bm-train	F10	1	0.4494133	2	1
F11	bm-train	F11	0	0.52353615	2	1
F12	bm-train	F12	1	0.46268475	2	1
F13	bm-train	F13	1	0.46502808	2	1
F14	bm-test	F14	1	0.34902862	1.5	1
G1	bm-train	G01	0	0.35349941	2	1
G2	bm-test	G02	1	0.47178266	2	1
G3	bm-test	G03	0	0.37860858	2	1
G4	bm-test	G04	1	0.36001387	3	1
G5	bm-test	G05	1	0.34992132	2	1
G8	bm-test	G08	1	0.48323873	2	1
G13	bm-train	G13	1	0.58676285	2	1
G14	bm-test	G14	1	0.56484234	2	1
H2	bm-train	H02	1	0.43237412	2.5	1
H6	bm-train	H06	0	0.47654739	2	1

H7	bm-test	H07	1	0.34227702	2	1
H9	bm-train	H09	0	0.31646851	2	1
H10	bm-test	H10	0	0.67290008	2	1
H11	bm-train	H11	0	0.43840089	1.5	1
H12	bm-test	H12	1	0.42841291	2	1
H13	bm-test	H13	1	0.4325819	2	1
H14	bm-test	H14	1	0.42672214	2	1
I1	bm-test	I01	0	0.50265795	2	1
I3	bm-train	I03	1	0.47241309	2	1
I4	bm-train	I04	1	0.3911947	2	1
I5	bm-test	I05	1	0.50575948	1.5	1
I6	bm-test	I06	1	0.39444658	2	1
I7	bm-test	I07	1	0.41271818	2	1
I8	bm-train	I08	1	0.41984674	2	1
I9	bm-test	I09	1	0.46695069	2	1
I11	bm-test	I11	1	0.47713417	2	1
I12	bm-train	I12	1	0.31550247	2	1
I13	bm-test	I13	0	0.58113855	2	1
I14	bm-train	I14	1	0.3111777	2	1
J1	bm-test	J01	0	0.33670238	2	1
J3	bm-test	J03	1	0.45433289	2	1
J4	bm-train	J04	1	0.36456752	2	1
J5	bm-train	J05	0	0.65112031	2	1
J6	bm-test	J06	0	0.47777674	2	1
J7	bm-test	J07	1	0.47147557	2	1
J9	bm-train	J09	1	0.41671023	2	1
J10	bm-train	J10	0	0.5385474	2	1
J11	bm-test	J11	0	0.546125	2	1
J12	bm-train	J12	1	0.55379772	2	1
J13	bm-train	J13	1	0.47003463	2	1
J14	bm-test	J14	1	0.44989935	2	1
A10					1	0
B14					1	0
D4					1	0
F6					1	0
H4					1.5	0
A8					2	0
B8					2	0
D5					2	0
D9					2	0
D12					2	0
E3					2	0

E7	2	0
G6	2	0
G7	2	0
G9	2	0
G11	2	0
G12	2	0
H1	2	0
I2	2	0
I10	2	0
J8	2	0
B1	2.5	0
G10	2.5	0
H8	2.5	0
H3		
H5		
J2		
